# Estimation of CYP3A4*1B single nucleotide polymorphism in patients with recurrent Major Depressive Disorder

**DOI:** 10.1002/mgg3.669

**Published:** 2019-04-25

**Authors:** Rafał Świechowski, Agnieszka Jeleń, Marek Mirowski, Monika Talarowska, Piotr Gałecki, Jacek Pietrzak, Damian Wodziński, Ewa Balcerczak

**Affiliations:** ^1^ Laboratory of Molecular Diagnostics and Pharmacogenomics, Department of Pharmaceutical Biochemistry and Molecular Diagnostics Medical University of Lodz Lodz Poland; ^2^ Department of Adult Psychiatry Medical University of Lodz Lodz Poland

**Keywords:** cytochrome P450, Major Depression, pharmacogenetics, polymorphism

## Abstract

**Background:**

Major depression is the most common mental illness in the world. Failures in treatment may occur due to the presence of a subtype of depression called TRD (Treatment‐ Resistant Depression). *CYP3A4* polymorphism (rs2740574) can increase the activity of Cytochrome P450 3A4, contributing to faster metabolism of xenobiotics and reduced response to treatment. The aim of the study was to assess the distribution of CYP3A4*1B in study and control group and to estimate the influence of particular genotypes on parameters such as: age at onset, severity of symptoms before treatment and on the effectiveness of therapy.

**Methods:**

Total of 192 patients were enrolled in this study (102 patients suffering from recurrent Major Depression Disorder, 90 healthy blood donors). PCR Restriction Fragment Length Polymorphism method with *MboII* enzyme was performed. The presence of CYP3A4*1B allele was evaluated on the basis of agarose gel electrophoresis.

**Results:**

There was a tendency in frequency of genotypes distribution in the study group in comparison with the control group (*p *= 0.050). There were no statistically significant differences in the distribution mutant allele among these two groups, but there was a tendency for mutant allele to occur more often in the study group (*p *= 0.050). No significant correlations were found between the specific genotype and the studied parameters: age at onset (*p *= 0.232), severity of the symptoms (*p *= 0.946), and efficacy of treatment (*p *= 0.882).

**Conclusion:**

The study suggests that CYP3A4*1B polymorphism have no influence on the predisposition to depression, the severity of depressive symptoms and the efficiency of antidepressant therapy.

## 1 INTRODUCTION

Over the last decades, there was a significant development in epidemiological research on depression. These studies have shown a considerable increase in major depression incidence. Data from recent years, show that about 6% of the world population suffers from depression (Rybakowski, 2016). It is expected that in 2020, depression will be the second illness, immediately behind ischemic heart disease, causing the feeling of disability by patients (Lecrubier, 2001).

Etiopathogenesis of depression is not fully understood. It is known that the occurrence of this illness results from a combination of genetic predisposition, environmental factors, physical condition, and the influence of stress. Twins studies have revealed that the heritability of the disease was at the level of 40%–60% for monozygotic twins and 25% for dizygotic twins. Those disproportions prove a significant contribution of genetic factors in the development of the disease (Kiyohara & Yoshimasu, 2009).

Pharmacological treatment is a method of choice to combat symptoms of clinical depression. Among clinicians, the most commonly prescribed medications are antidepressants which are a large group of drugs divided in terms of chemical structure and mechanism of action. From a wide group of pharmaceuticals: tricyclic antidepressants (TCAs), monoamine oxidase inhibitors (MAOIs), selective serotonin reuptake inhibitors (SSRIs), norepinephrine and dopamine reuptake inhibitors (NDRIs), dual serotonin and norepinephrine reuptake inhibitors (SNRIs) can be mentioned (Penn & Tracy, 2012). Unfortunately, determination of appropriate treatment scheme, requires time and observation from both, patient and psychiatrist, because the first drug therapy has positive efficiency only for 50% of patients (Sutherland, Sutherland, & Hoehns, 2003). There are many reasons for the failure of drug therapy. The main reason is the type of depression, called Treatment‐Resistant Depression (TRD). This term describes Major Depressive Disorder (MDD) that do not respond to at least two, properly chosen antidepressants used for at least three weeks. Causes of the phenomenon of drug resistance may be as follows: neurotic personality traits, positive family history, wrong diagnosis, coexistence of somatic and other mental diseases, wrong treatment scheme, changes in the pharmacokinetics and pharmacodynamics. The cytochrome CYP3A4 polymorphism may be responsible for the pharmacokinetic factor (Bogdanowicz & Kalinowski, 1992).

Cytochrome p450 (CYP) is the basic component of the mixed function oxygenases (MFO) which is responsible for biotransformation of endogenous compounds (Navrátilová, Paloncýová, Berka, & Otyepka, 2016). This class consists of more than 50 genes, but one of the most important is *CYP3A4 *(OMMIM 124010), which is involved in biotransformation of more than 50% of drugs currently used. Cytochrome P450 3A4 is mainly located in liver and small intestine. CYP3A4 is most plentiful cytochrome in those two organs (Ali, Al‐Azhary, & Mokhtar, 2014; Lynch & Price, 2007). *CYP3A4* gene is located on chromosome 7q21.3‐q22.1, it has a length of 27,952 base pairs and consists of 13 exons (Keshava, McCanlies, & Weston, 2004). In a wide range of metabolized xenobiotics by CYP3A4, we can also find different groups of antidepressants (Table 1) (Ayano, 2016). In the study of cytochrome p450 3A4 many allelic variants of this gene were detected. Conversion of adenine to guanine at nucleotide 392 in the promoter sequence of the gene (A392G), produces mutant allele CYP3A4*1B. It is a promoter region mutation causing the increase in cytochrome P450 3A4 enzymatic activity by increasing its expression (Zochowska, Wyzgał, & Paczek, 2012). The presence of a mutant allele is varied in different populations. Allele is not present in Asian population, while in African Americans population the prevalence is about 45%–66.7%. For the Caucasian population, the prevalence is about 4.5%–9.6% (Wojtczak & Skrtętowicz, 2009).

**Table 1:** Antidepressants metabolized by CYP3A4 (Ayano, 2016)

Antidepressants group	Drug name
TCAs	Amitriptyline, imipramine, lomipramine, mianserin
SSRIs	Citalopram, escitalopram, paroxetine, fluoxetine
SNRIs	Venalafaxine, trazodone
Other antidepressants	Buspirone, reboxetine nefazodone, mirtazapine

Note

SNRIs: serotonin‐norepinephrine reuptake inhibitors; SSRIs: serotonin–norepinephrine reuptake inhibitors; TCAs: tricyclic antidepressants.

The aim of the study was to evaluate the distribution of CYP3A4*1B allele in patients suffering from recurrent Major Depressive Disorder, compared to the distribution in the control group and to assess the influence of particular genotypes on parameters such as: age at onset, severity of symptoms before treatment and on the effectiveness of pharmacotherapy.

## 2 MATERIAL AND METHODS

### 2.1 Material

The study included 102 DNA samples, isolated from peripheral blood of patients, diagnosed with recurrent MDD. The study group comprised of 67 females and 35 males between the age 18 and 63 years with an average age of 48 years and average age at onset, 42 years. Patients were diagnosed by psychiatrists and included into the study with ICD‐10 criteria (F32.0‐7.32.2; F33.0‐F33.8). The severity of depressive symptoms was assessed using a Hamilton Rating Scale for a Depression (17 questions version). Pharmacotherapy was conducted using drugs such as: SSRIs, mirtazapine, quetiapine, mianserin, acid valproic, venlafaxine, trazodon, levomepromazine, agomelatine, and doxepin. Drugs were dosed individually or in combination. The control group consisted of 90 healthy blood donors (55 women and 35 men) who were selected randomly. Their age ranged from 20 to 50 years with an average age of 35 years.

The study was conducted in accordance with the Declaration of Helsinki and was approved by the Bioethics Commission (RNN / 566/08 / KB).

### 2.2 DNA isolation

DNA isolation was performed in accordance with the protocol “Blood Mini” (A&A Biotechnology). Isolated DNA was stored at −20°C until further analysis.

### 2.3 Polymerase chain reaction

**Figure 1:**
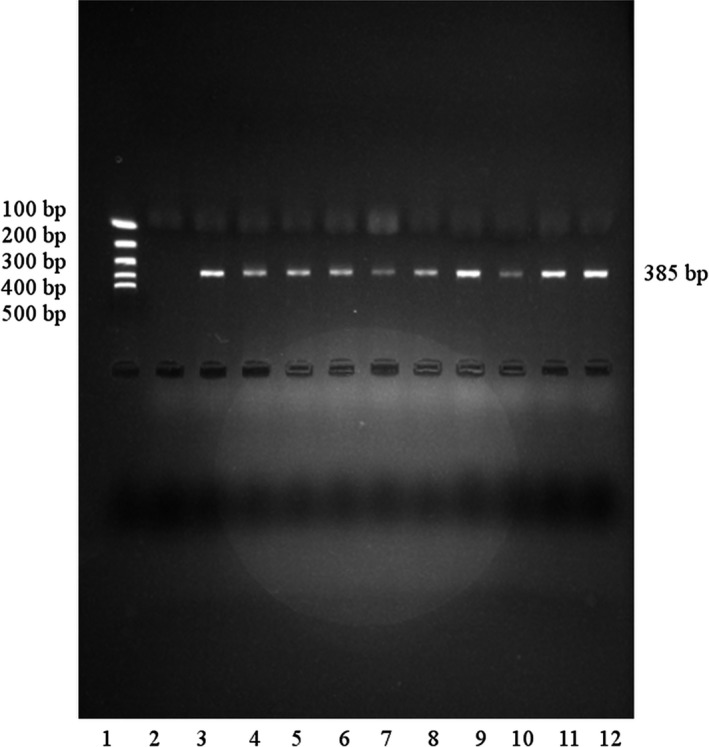
Agarose gel electrophoresis of *CYP3A4* gene. Lane 1—molecular marker, Lane 2—negative control, Lanes 3–12—positive samples

The primers used were as follows F: GGA ATG AGG ACA GCC ATA GAG ACA AGG GGA, R: CCT TTC AGC TCT GTG TTG CTC TTT GCT G. Reaction components: 1 µl of isolated DNA, 0.4 µl (10 µM) of primers (F and R), 0.5 µl (25 mM) MgCl_2_, 1.2 µl (10 mM) dNTP, 2.5 µl (DreamTaq™ Buffer, Thermo Scientific™), 18.7 µl H_2_O, 0.3 µl (500U) (DreamTaq™ DNA Polymerase, Thermo Scientific™). After 5 min of initial denaturation at 98°C, 30 cycles of 60 s at 95°C, 90 s at 60°C, and 120 s at 72°C. The final elongation proceeded at 72°C and lasted 10 min. PCR product with a size of 385 base pairs was obtained, electrophoresis results are presented in Figure 1. PCRs were carried in (MJ Mini Thermal Cycler, Biorad).

### 2.4 RFLP detection of rs2740574 polymorphism (NG_008421.1:c.392A > G)

**Figure 2:**
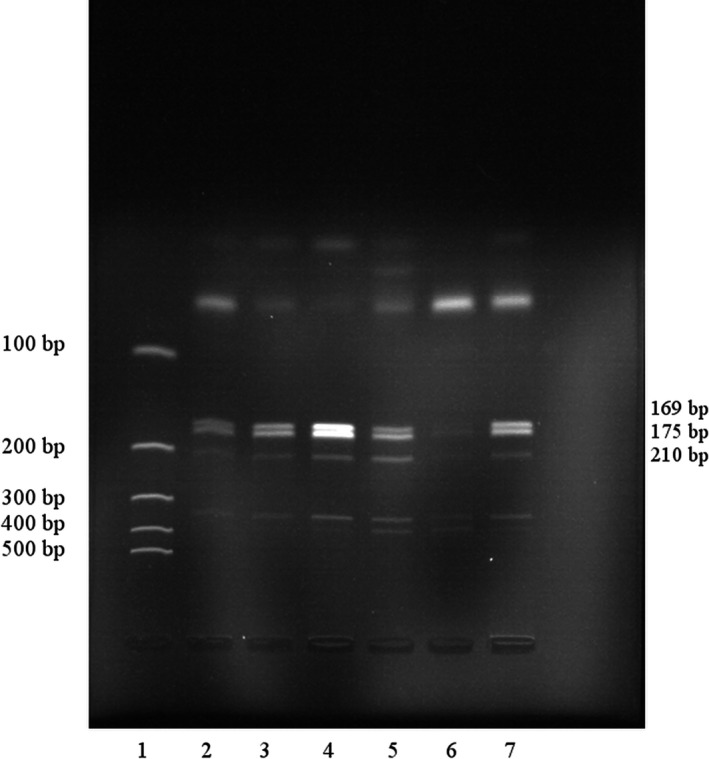
Agarose gel electrophoresis of *CYP3A4 *PCR‐RFLP digested with *MboII *enzyme. Lane 1—molecular marker, Lane 2–5,7—genotype AG

Digestion of PCR product, was performed using the *MboII *enzyme (*Moraxella bovis*, EURx, Poland, 10 U/µl). A volume of 0.1 µl of restriction enzyme was added in a total reaction volume of 20 µl and incubated at 37°C for 17 hr. Homozygous wild‐type patients (genotype AA) produced 175, 169, and 41 bp fragments. Homozygous for mutant type (genotype GG) produced 210 and 175 bp fragments. Heterozygous variants (genotype AG) showed the presence of 210, 175, 169, and 41 bp fragments. The CYP3A4*1B polymorphism was assessed on the basis of 4% agarose gel electrophoresis. An exemplary result of digestion is shown in Figure 2.

### 2.5 Statistical analysis

Chi‐square was used to analyze the deviance from HWE and to analyze the difference in distribution of CYP3A4*1B allele among study and the control group. The Student’s *t* test and the Mann–Whitney U test were used to analyze the individual characteristics of the study group. Statistical analysis was performed using STATISTICA 13 (StatSoft Inc.). *p* < 0.050 was measured statistically significant.

## 3 RESULTS

Genotype and allele distribution for *CYP3A4 *polymorphism (rs2740574) and their association with Hardy–Weinberg equilibrium (HWE) are given in Table 2. In the study group, the AG genotype was most frequently observed (84, 82.4%), mutant homozygote GG occurred in 18 patients (17.6%). No AA wild‐type homozygote was found in the study group. There was statistically significant deviance from HWE for the study group (χ^2^ = 49.98, *p* < 0.001) and for the control group (χ^2^ = 33.79, *p *< 0.001).

**Table 2:** Distribution of CYP3A4*1B genotype, alleles in the study and control group and their association with Hardy–Weinberg equilibrium

Variables	Genotyping	Study group (%)	Control group (%)
		*N* = 102	*N* = 90
CYP3A4*1B	AA	0 (0)	5 (5.6)
	AG	84 (82.4)	72 (80.0)
	GG	18 (17.6)	13 (14.4)
	A	0.41	0.46
	G	0.59	0.54
Deviation from HWE	χ^2^	49.98	33.79
	*p*‐value	*p* < 0.001	*p* < 0.001

Note

HWE: Hardy–Weinberg equilibrium.

There was a tendency in the frequency of genotypes distribution in the group of patients suffering from depression in comparison to the control group (*p* = 0.050). There were no statistically significant differences in the distribution of G and A allele among these two groups, but there was a tendency for G allele to occur more often in the study group (*p* = 0.050). The investigated characteristics were then compared with the individual genotypes of the study group.

At first, the correlation between age at onset and genotype was investigated. In homozygous GG patients group, median age at onset was lower by 8 years in comparison to heterozygous AG group, but this difference was not statistically significant (*p* = 0.232). The mean values for the severity of symptoms measured before the treatment were very similar in the homozygous and heterozygous group (*p* = 0.946). Statistical analysis was also performed for treatment effectiveness in patients with different genotypes. The effectiveness of treatment was expressed as a difference in Hamilton Depression Rating Scale score assessed before and after patient treatment. No statistically significant differences were found (*p* = 0.882). Demographic information and selected variables of the study group are summarized in the Table 3.

**Table 3:** Demographic and selected variables of the study group

Variables	Study group *N* = 102			*p*
	GG (*N* = 18)	AG (*N* = 84)	All	
Age, years (median)	47,5	51	51	
Age at onset (median)	37	45	44	0.2322
HDRS (*M* ± *SD*)	21.94 ± 7.72	21.82 ± 7.16	21.83 ± 7.22	0.9456
HDRS change (*M* ± *SD*)	15.33 ± 5.96	15.07 ± 6.93	15.12 ± 6.74	0.8818

Note

HDRS—Hamilton Depression Rating Scale score before treatment, HDRS change—difference in Hamilton Depression Rating Scale score assessed before and after patient's treatment.

## 4 DISCUSSION

Studies on cytochrome p450 enzymes revealed the occurrence of numerous mutations and the presence of many polymorphic variants of genes coding those enzymes. Some of them are responsible for the excessive metabolism of xenobiotics and partially explain the low effectiveness of pharmacotherapy. Identifying a patient's genotype can have a fundamental impact on choosing the right dose of drug (Staddon, Arranz, Mancama, & Kerwin, 2002).

The aim of the study was to evaluate the CYP3A4*1B polymorphism in the group of patients suffering from recurrent MDD and to compare it to the control group consisting of healthy blood donors. The impact of individual genotypes on the parameters: age at onset, severity of symptoms before treatment and pharmacotherapy efficacy, was also checked. There were no statistically significant differences in the distribution of genotypes between the study and control groups. No significant correlations were found between the specific genotype and the studied parameters. Probably the analysis for CYP3A4*1B were performed for the first time in the group of patients suffering from depression, therefore it is not possible to compare them with other data.

Both the study and the control group showed a statistically significant deviance from HWE. The deviation from HWE is manifested by too many heterozygotes in both control and study group. The deviation is smaller in control group. There are few possible causes for this case: genotyping error, population is too small, and there is a difference in allele distribution between the gender, random sampling error, individuals with a certain genotype have a higher probability to have been chosen (Waples, 2015). Some samples were randomly replicated to exclude genotyping errors and the results were the same. The chi square Pearson test did not show any statistically significant differences in the distribution of genotypes and alleles relative to gender (*p* = 0.520). The most likely cause is a random sampling error or, in the case of the study group, a tendency to pick up heterozygous individuals.

Despite the *p* score of 0.050, the differences between the genotype distribution in two groups was not defined as a statistically significant one. There was a tendency for genotype AG and GG to occur more often in the study group than in the control group, and tendency for CYP3A4*1B allele to occur more often in the study group. No wild homozygotes were found in the study group as opposed to the control group. It can suggests that people suffering from recurrent MDD are more likely to have CYP3A4*1B. It can also be the reason why the deviance from HWE was higher in the study group.

The significance of CYP3A4*1B polymorphism was tested in multiple disease units. However, there were not any study about Major Depression. In research about acute myeloid leukemia (AML), Ali et al. showed that heterozygous genotype AG was statistically significant more common in patients with AML (21.6%) than in the control group (2.8%). In our study, heterozygous AG had a similar high rate in the study (82.4%) and the control group (80.0%) (Ali et al., 2014).

In the study from 2007, there was no correlation between the CYP3A4*1B allele and the incidence of prostate cancer in the Portuguese population. The percentage of AG genotypes in the study group was also much lower than in this work. In the test group (*n* = 443), the AG genotype was present in 10% of cases and in the control group (*n* = 337) in 9%. The selection of CYP3A4*1B polymorphism was correct because testosterone, which is associated with prostate cancer, is metabolized by CYP3A4 cytochrome (Nogal et al., 2007).

In the research conducted by a team of Bangladeshi researchers, the impact of CYP3A4*1B polymorphism on cervical cancer susceptibility was tested. There were no statistically significant differences in the distribution of genotypes between the study and the control group. This study was performed on a small group of patients (*n* = 30) so this could be a reason why the association was not found. The authors pointed to the lack of access to tumor tissue as one of the limitations of the study. However, it should be noted that the object of their study was a polymorphism of the gene, and not the expression. It could be crucial for the assessment of *CYP3A4* gene expression (Abdullah, Shafayat, Saifuzzaman, Fahim, & Golam, 2016).

Similar studies were conducted for other diseases from the mental illness group. In the work on neuroleptic drug resistance in schizophrenic patients, correlation between CYP3A4*1B gene polymorphism, and the effectiveness of neuroleptic treatment was reported. Homozygotes were more commonly classified as resistant to treatment compared to heterozygous subjects (Kohlrausch et al., 2008). There were no statistically significant differences in effects of treatment for patients with various genotypes in the following work. In patients with schizophrenia, the AA genotype was most common, and homozygous GG was not found (Kohlrausch et al., 2008). The results from the study on schizophrenia patients differ significantly from those presented above. In patients with depression most common was genotype AG, wild homozygotes was not observed.

Research conducted in 2013 demonstrated that there is a correlation between CYP3A4*1B polymorphism and the drug used in the treatment of epilepsy—carbamazepine. The study group consisted of 90 patients suffering from epilepsy who were treated with carbamazepine. The study group was not ethnically homogeneous, white Americans, and African Americans participated in the project. The study showed that the presence of mutated CYP3A4*1B allele may be associated with the decreased clearance of carbamazepine (Puranik et al., 2013). Because of the significant differences in the occurrence of the mutant allele between ethnic groups, the study should be conducted in patients with similar ethnic backgrounds. Ethnic differences may have a significant impact on the outcome of this research. Review article summarizing 37 researches from MEDLINE® suggest that current evidence does not support the use of Cytochrome P450 genotyping to guide SSRI treatment of patients suffering from depression. CYP450 polymorphism could be one of many features affecting response to antidepressant treatment. Despite many studies on the metabolism of antidepressants, there is still a low number of researches on genotyping as an important tool in depression therapy. At this moment, more research is needed. Genotyping in the area of mental illness is only a scientific method rather than a clinical practice (Thakur et al., 2007).

Study performed on patients suffering from recurrent MDD, shows that majority of them had AG genotype. The results differ significantly from the existing data in the scientific literature, where the mutant CYP3A4*1B allele is very rare. In the control group, the CYP3A4*1B allele was less frequent, but the difference between the literature data, which reported that the mutant allele is present only in 9% of Caucasian patients, is very high. Such differences in the control group may occur due to the difference in the group size or a random sampling error. In the case of the study group, differences may occur due to similar causes. It is possible that there is a link between the occurrence of depression and the genotype containing the CYP3A4*1B mutant allele. This association can be supported by a tendency for the increased occurrence of this allele in the clinical group. There are not many scientific reports with which we can compare this results. It is hard to collate two different disease units in which different pharmaceutics are used. There was no statistically significant association between the subjects of research and the specific genotype. The lack of correlation between the genotype and the effectiveness of treatment may be due to a very wide range of drugs commonly used in combination in the study group. In addition, the specific isoenzyme can metabolize drugs used in other disease unit more efficiently. Therefore, it can be a reason why there are reports of a relationship between the efficacy of the treatment and the genotype in other disease units. It was expected that the response for treatment of patients with CYP3A4*1B allele would be weaker due to the increased enzyme activity and faster drug metabolism.

There are too few analyses of the correlation between gene polymorphism and depression. This is the first study of CYP3A4*1B polymorphism performed on a group of people suffering from depression. This work cannot be compared to other research. A mutant allele was found in all subjects with depression. This may indicate a certain association of CYP3A4*1B polymorphism with recurrent MDD. To confirm this theory, further research in this area is recommended.

## CONFLICT OF INTEREST

The authors report no conflicts of interest.

## AUTHOR CONTRIBUTION

Rafał Świechowski—manuscript preparation, experiment conduct, data analysis. Agnieszka Jeleń—data analysis. Marek Mirowski—critical revision. Piotr Gałecki—Study conception and design, collecting samples, diagnosis. Monika Talarowska—Collecting samples and written permission, diagnosis. Jacek Pietrzak—experiment conduct. Damian Wodziński—experiment conduct. Ewa Balcerczak—study conception and design, critical revision.
